# Advanced breeding techniques in *Brassica juncea* L. for sustainable production under changing climate

**DOI:** 10.1080/15592324.2026.2648963

**Published:** 2026-04-04

**Authors:** Ashita Pawaiya, Rajesh Kumar Pradhan, Atul Kumar Johri, Meenakshi Dua

**Affiliations:** a Microbial Ecology Laboratory, School of Environmental Sciences, Jawaharlal Nehru University, New Delhi, India; b School of Life Sciences, Jawaharlal Nehru University, New Delhi, India

**Keywords:** *Brassica juncea*, crop improvement strategies, abiotic and biotic stresses, microbial symbionts

## Abstract

*Brassica juncea* (L.) Czern. & Coss. is an agronomically important crop cultivated worldwide as a valuable source of oil. It is a major source of edible oil in South Asia because of its high oil content, nutraceutical value, and balanced fatty acid contents. In addition to being considered a relatively hardy crop with high economic value, its productivity potential is restricted by susceptibility to various biotic and abiotic stresses, including diseases, pests, drought, heat, frost, and salinity. These constraints adversely affect yield, often leading farmers to move towards alternative crops. In this context, the use of advanced genomics, transcriptomics, and proteomics approaches can provide molecular insight into the evolutionary history, genetic diversity and adaptive response of *B. juncea* under stress and at different developmental stages. A comprehensive understanding of its molecular architecture and advanced crop improvement strategies will culminate in the development of high-yielding, stress-resistant cultivars, facilitating sustainable mustard production under a changing climate.

## Introduction

*Brassica juncea,* commonly known as Indian mustard, is a highly diverse and agronomically important crop. It is originated from natural interspecific hybridization of *B. rapa* and *B. nigra* and belongs to the family Brassicaceae.^1^ It is further classified into four sub species such as *juncea* (seed mustard), *integrifolia* (leaf mustard), *napiformis* (root mustard), and *tumida* (stem mustard).^2^ *B. juncea* has demonstrated remarkable ecological adaptability and spreads across Asia, Africa, Europe, Australia, and America.^3^ Genome-wide sequence studies have revealed that West Asia as the center of origin of *B. juncea*. Large-scale genome and germplasm assemblies provide insight into the domestication, diversification and artificial selection of favorable traits such as genes associated with morphology, flowering time, seed weight, and adaptive responses.^4^ *B. juncea* is an allotetraploid (AABB, 2n = 36), a branched plant with stalked, broad, and pinnatifid leaves. The stem grows upto 75  cm, and the pods are elongated and narrow with black or yellowish-white seeds.^5^ *B. juncea* is predominantly cultivated in India for edible oil due to its high oil content (38%–42%), accounting for approximately 27% of total oilseeds and 31% of vegetable oil production.^6^ *B. juncea* covers approximately 90% of the total cultivable area under rapeseed- mustard crop cultivation.^7^ Additionally, mustard is the most important crop for small and marginal farmers because it is used as an oilseed crop as well as a condiment and medicine. It is also used in industries such as the tanning industry for softening leather. A higher protein content limits the ability of seed meal to produce good manure and cattle feed because of the presence of high amounts of glucosinolates.^8^ Its oil is a good source of omega-3 fatty acids (MUFAs) and other fatty acids, such as linoleic acid and alpha-linoleic acid.^9^ The presence of flavonoids, phenolics, sterols, triterpene alcohols, and a balanced level of saturated and unsaturated fatty acids contributes to the nutraceutical properties of the oil. On the other hand, the presence of high erucic acid adversely affects health.^10^

**Figure 1.**
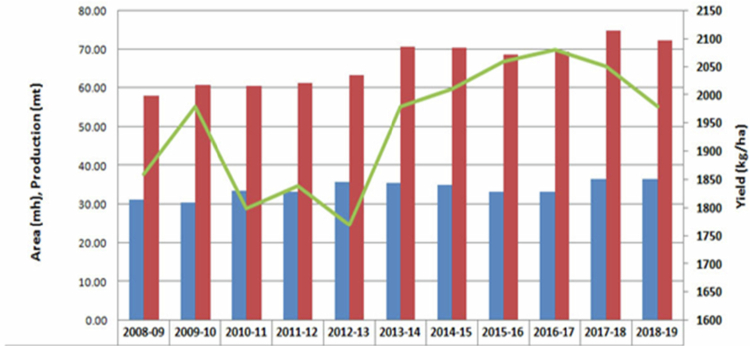
World rapeseed-mustard trends.

**Figure 2.**
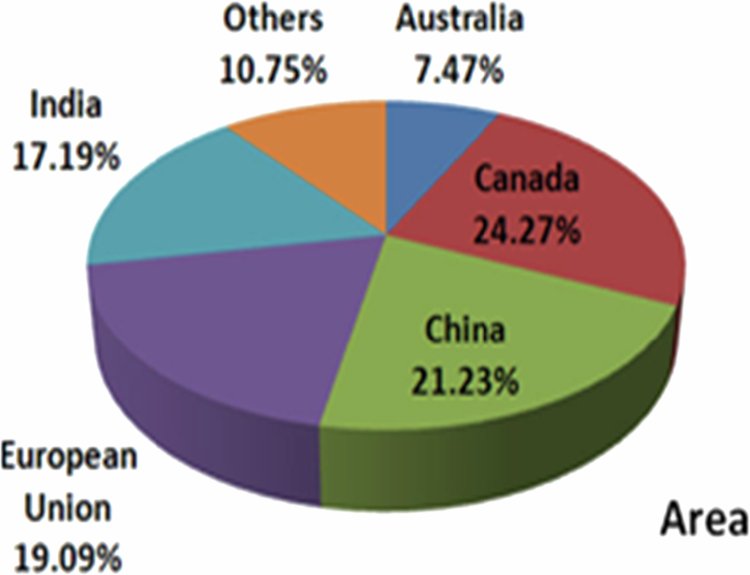
Contribution of different countries in rapeseed-mustard acreage during 2013–2014 to 2017–2018.

**Figure 3.**
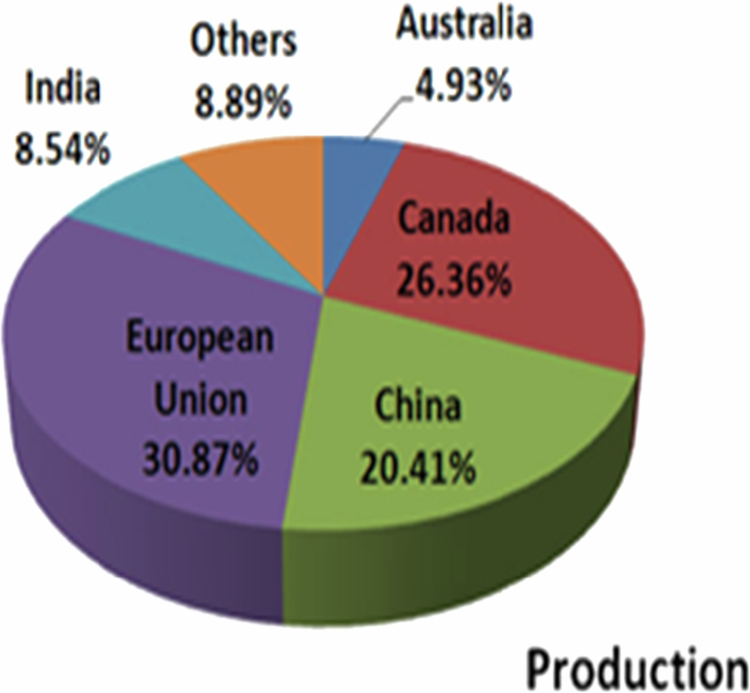
Contribution of different countries in rapeseed-mustard production during 2013–2014 to 2017–2018. Source: ICAR – Directorate of Rapeseed-Mustard Research (DRMR), 2019, India.

**Figure 4.**
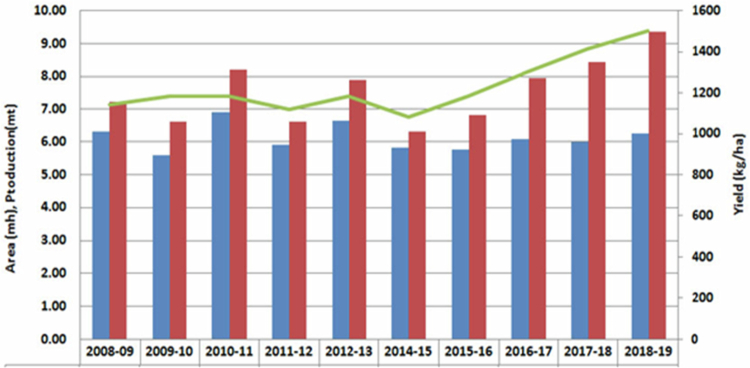
Agricultural land and yield under mustard cultivation in India. Source: ICAR – Directorate of rapeseed-mustard research.^13^

It has been established that a temperate and sub-tropical climate most suited for *B. juncea* cultivation because of its tolerance to 500–4200 mm precipitation, 6–27 °C annual temperature, and pH 4.3–8.3.^11^ Globally, there has been a rapid surge in the production of rapeseed-mustard from 2013 to 2019 (Figure 1). In 2013–2016, the area under mustard cultivation was highest in Canada (8 mha), followed by China (7 mha) and India (6 mha).^12^ In 2018–2019, globally, the total area under cultivation was 36.59 mha (ICAR-DRMR, 2019). The productivity during 2013–2016 was 3640 kg/ha in the European Union followed by 1161 kg/ha in India, while during 2018–2019 it was 1980 kg/ha in India (Figures 2 and 3). In India, rapeseed-mustard is grown in the North, North-western, and North-eastern region of the country and accounts for approximately 75%–80% of 6.23 mha agricultural land under the crops in India during 2018–2019 (Figure 4).^12^^,^^13^

The productivity of *B. juncea* is determined by various biotic stresses, such as clubfoot, Alternaria blight, stem rot, and aphid infection, as well as abiotic stresses, such as drought, frost, heat, and salinity. Improved cultivars of mustard have been reported to stabilize oil yield, enhance quality (low erucic acid and glucosinolate) and increase stress tolerance.^14^ This review provides insights into the understanding of crop development strategies, multi-omics and the molecular basis of stress tolerance and yield-related traits in *B. juncea* for the development of high-yielding and stress-resistant cultivars that are well adaptable to changing climate conditions.

***B. Juncea*** **cultivars:​​​​​ **The first Indian mustard hybrid, named “NRCHB506,” was developed at the Directorate of Rapeseed-Mustard Research, Bharatpur, Rajasthan state, which can catapult the output of the country’s key oil crop. Since the inception of the Mustard Research Program in India, a number of tolerant varieties to various abiotic and biotic stresses of rapeseed-mustard has been developed. The India Coordinated Research Project on Rapeseed-mustard (AICRP-RM) has released 248 varieties of RM, out of which 113 belong to *B. juncea*. These include hybrid varieties and varieties that are tolerant to white rust, alternaria blight, powdery mildew and some abiotic stresses, such as salinity and high temperature.^13^ The primary requirement for the cultivation of mustard is land and seedbed preparation. It has been suggested that mustard seedbeds should be firm, moist, and uniform, which allows good seed-to-soil contact, even at planting depth and quick moisture absorption leading to uniform germination.^11^ It has been observed that tillage affects both crop growth and grain yield. It has been suggested that minimum tillage, with or without straw, improves the soil water conservation and water availability during the crop growth, which results in the increase in root mass, yield components and seed yield.^15^ It is preferred for mustard because it retains more soil moisture in the early growing season. The subsequent release of conserved soil moisture regulates the appropriate plant water conditions, soil temperature, and reduces the mechanical strength of the soil, resulting in better root growth and higher mustard seed yield. Other critical features to obtain healthy crops include land preparation, seed germination, sowing of seeds and seedling growth, improved root development, early stem elongation, and rapid ground covering ability. In addition, early flowering occurs under the low-temperature and radiation regime, good, and healthy seeds should be selected to grow at the proper time to maintaining an optimum plant population. In this context, to improve the seed quality and germination, plants must be soaked in 0.025% aqueous pyridoxine hydrochloride solution.^11^ Further, sowing time is the most vital nonmonetary input to achieve target yields in mustards.^11^ Sowing at the optimum time results in higher yields due to the suitable environment that prevails at all growth stages. Though different varieties have a differential response to the date of sowing, mustard sown on 14 and 21 October presented significantly more days to 50% flowering and maturity compared to October 7 planting. Delayed sowing results in poor growth, low yield, and low oil content and also affects the incidence of insect-pest and disease.

It has been observed that mustard aphids, the most devastating pests, can be controlled if a crop is sown in the first week of November. Generally, sowing with ridge and furrow technique is superior to conventional flat sowing for the growth parameters of *B. juncea*. Another technique of transplanting is considered to save time and resources, as it reduces days to maturity and results in increased seed yield. Ridge transplanting reduced the amount of water applied by 30% for each furrow as compared to 45 cm row spacing in the flat method without any loss in seed yield. The crops should be distributed uniformly with an appropriate geometry for the efficient use of nutrients, moisture, and suppression of weeds, leading to high yield. In the case of *B. juncea*, the recommended spacing is 30 × 10, and for hybrids, it is 45 × 10 to avoid mutual shading between plants and competition among themselves. Some studies have shown that the productivity of Indian mustard can be increased considerably by incorporating crops into existing cropping systems. mustard can be intercropped successfully with wheat, barley, gram and lentil under rain-fed conditions and with potato under irrigated conditions. Intercropping systems such as potato + mustard (3:1), field pea + mustard (3:1) and gram + mustard (3:1) are widely practiced in mustard growing areas.^16^ In a rainfed area, the green manuring enhances the seed yielding of mustard when grown in combination with potato (1:3), mustard + wheat (1:5), mustard + barley (1:5) than pure mustard (Shekhawat et al., 2012). Some varieties are recommended by the Directorate of Oilseeds Development (DOD),^17^ Ministry of Agriculture and Farmers Welfare Govt. of India are listed in Table 1.

**Table 1.** Varieties of Indian mustard grown in India (Adapted from Directorate of Oilseeds Development, MInistry of Agriculture and Farmers Welfare, Govt. of India, 2025).^18^

Variety/hybrid	Year of release	Yield potential (kg/ha)	Oil content (%)	Recommended states/region/situations	Specific features/traits
RLC 1 (ELM 079)	2007	1600–2000	38	Punjab	Low erucic acid
Pusa Mustard-21	2007	2111	34.0–40.0	Delhi, Haryana, J&K (Plains) Punjab, Rajasthan, Western Uttar Pradesh	Low erucic acid (<2%)
Shatabdi (ACN 9)	2007	468–1291	32–40	Maharashtra	Suitable for timely and late sown conditions
TPM 1	2007	1127–1682	34–39	Maharashtra	Yellow seeded
Pusa mustard 22 (LET 17)	2008	2007	35.5	Haryana, Punjab, Jammu, parts of Rajasthan and Delhi	Suitable for Irrigated conditions, low erucic acid variety
Pusa Vijay (NPJ 93)	2008	1870–2715	35–41	Delhi	High temperature tolerant at seedling stage and salinity
CS 56 (CS-234-2)	2008	1170–1423	34.2–38.0	Haryana, Punjab and parts of Rajasthan	Suitable for late sown conditions, salt tolerant, 1000 seed weight more than 6 g.
Pusa mustard-24 (LET-18)	2008	1241–2904	32.0–39.7	Haryana, Punjab, New Delhi and parts of Rajasthan	Low erucic acid (<2%)
Dhara mustard hybrid 1 (DMH 1) Hybrid	2009	1782–2249	38–42	Delhi, Haryana, Punjab, J&K and Rajasthan	High pod density, resistant to white rust
NRC HB 101	2009	1382–1491	35–42	Madhya Pradesh, Uttar Pradesh, Uttarakhand and Eastern Rajasthan	Suitable for late sown irrigated conditions
NRCHB506 (Hybrid)	2009	1550–2542	39–43	Madhya Pradesh, Uttar Pradesh, Uttarakhand and Eastern Rajasthan	High adaptation
Pusa Tarak	2009	1852–1996	38–42	Delhi	High temperature tolerant and bold seeded
Pusa mustard 25 (NPJ 112)	2010	1324–1654	36–41	Delhi, Haryana, Punjab, Jammu & Kashmir, Rajasthan, Western Uttar Pradesh	Suitable for early sown irrigated conditions, high temperature tolerance at juvenile stage
Pitambari (RYSK-05-02)	2010	1536		Central	U.P., West Bengal, Haryana, Bihar, Gujarat, Uttarakhand and Manipur. Early maturity 110–115 d.
Pusa Mustard 26 (NPJ 113)	2011	1481–1895	30–41	J&K, Punjab, Haryana, Rajasthan, Delhi & U.P.	Suitable for late sown irrigated conditions in rabi season.
Pusa Mustard 27 (EJ 17)	2011	1437–1659	40–45	U.P., M.P., Uttarakhand & Rajasthan	Suitable for early sown irrigated conditions & for multiple cropping
Pusa Mustard 28	2012	1912–2098	40–42.8	Delhi, Haryana, J&K, Punjab, Rajasthan	Early sown
RLC-2 (ELM 123)	2011/2012	2039–2342	36.3–38.9	Delhi, Haryana, Punjab, Jammu, Rajasthan	Superior oil quality
DRMRIJ-31	2013	2225–2750 kg/ha	39– 42.6	Delhi, Haryana, Jammu & Kashmir, Punjab and parts of Rajasthan	Timely sown irrigated conditions
DRMR 150-35	2020	1200–1800 (kg/ha)	36.7–42.8	Orissa, WB, Bihar, Jharkhand, Chhattisgarh and Assam	Rainfed condition
DRMR 1165-40	2020	2200–2600 (kg/ha)	41.2	Jammu, Punjab, Haryana, Delhi and Rajasthan	Rainfed, timely sown
DRMRIC 16-38	2021	1733 (kg/ha)	37.6–40.9	Jammu, Punjab, Haryana, Delhi and northern Rajasthan	Late sown rainfed condition
DRMR 2017-15	2021	1686–1847 (kg/ha)	40.7	Jammu, Punjab, Haryana, Delhi and parts of Rajasthan	Late sown irrigated condition/high seed yield & oil yield
BPM 11	2024	1859 (kg/ha)	37.8	Rajasthan, Uttar Pradesh, Madhya Pradesh, Uttrakhand and Bihar	Late sown condition. White rust resistant
BPMQ 47	2025	2348 (kg/ha)	40.5	Jammu, Punjab, Haryana, Delhi and Rajasthan	Timely sown irrigated conditions. White rust resistant Low erucic acid
DRMR 2018-25	2025	2596 (kg/ha	40.6	Jammu, Punjab, Haryana, Delhi, Rajasthan and Western Uttar Praedsh	Timely sown irrigated conditions White rust resistant
DRMRCI (Q) 172 (BPM Q 172)	2025	2304 (kg/ha)	41.8	Rajasthan, Uttar Pradesh, Madhya Pradesh, Uttarakhand, Bihar	Timely sown irrigated conditions. Low erucic acid White rust resistan

### Impact of biotic and abiotic stresses

*B. juncea* is cultivated under a wide range of ecological diversities, including irrigated/rainfed soil, salt-affected soil and a wide range of agro-climatic conditions providing edible oils, condiments, and animal feeds. However, accelerated anthropogenic climate change has hampered the yield potential of crops by using abiotic and biotic stresses. Abiotic stress is the effect of environmental changes such as drought, frost, salinity, metal toxicity, and heat stress in crops. Biotic stress is the result of damage caused to plants by other living organisms, such as bacteria, fungi, parasites, insects, weeds, and native plants. *B. juncea* is highly susceptible to a number of diseases and pests under this stress, Alternaria blight, white rust, and Sclerotinia rot are some important disease and aphids, mustard saw flies, and painted bugs are some important insects that significantly affect the growth and development of crops, resulting in pre and post harvesting losses.

### Abiotic stresses

Globally, abiotic stress is the key source of crop loss, reducing more than 50% the average yields for most major crop plants.^19^ Many abiotic stresses directly or indirectly affect brassica crops, as they are mainly cultivated in semi-arid or arid areas that are prone to drought and salt stress. It is grown at an optimum temperature of 15–22 °C, but temperature above 35 °C and below 10 °C may causes injury to reproductive organs at developmental stages. This stress adversely affects agro-morphological, disturbing the normal growth and yield of crops and influences the important morpho-biochemical characteristics of crops, such as root and shoot length, shoot fresh and dry weight, proline content, relative water content, chlorophyll content, antioxidant enzymes activity and cell injury, by disturbing normal oxidative processes in brassica species.^20^ In many cases, abiotic stress is primarily unavoidable and becomes a harmful factor concerning the growth and productivity of *Brassica*. Generally, plants have the capacity to acclimatize, resulting in physiological adjustments in plants that protect them from injury/impaired growth under extreme environmental conditions. It has been reported that *Brassica* species of tropical and subtropical origins are sensitive to chilling (0–15 °C) and freezing (<0 °C) stress and lack the mechanism of cold acclimation.^21^ It can be managed by exploring genetic resource material and agronomic factors.^22^ The development and cultivation of various improved varieties that are tolerant to stress can reduce the production loss of crops. Various crop improvement techniques (molecular approach) such as marker-assisted selection and the identification of quantitative trait loci (QTL) for drought, salt, heat stress, and frost help in improving tolerance potential of mustard.

### Drought

Increasing anthropogenic interventions accelerates the climatic changes that make drought a considerable threat to agricultural production. Drought can adversely affect plant growth at various stages, from seed germination to reproduction and flowering to harvesting, and ultimately results in oil and yield penalty.^7^ In *B. juncea*, which strongly impacts on emergence, growth, quantity and quality of produce through phenological, physiological, and biochemical pathways.^23^ Phenological steps such as seedling establishment degrade because of the uneven distribution of *Brassica* seeds, which occur mainly during drought stress. Physiological changes in the water potential and water content of water-stressed leaves through osmoregulation are shown in *B. juncea*. It has been observed that the most sensitive stage for drought injury is flowering, which results in high loss in seeds as well as oil yield by 29.5% and 31.7%, respectively.^24^ Drought decreases the seed oil content and yield of *B. juncea* at a rate of 2.6% and 25%, respectively, due to a significant loss in the number of seeds per number of siliquae per plant and loss in test weight of the seeds.^25^ It also reduces chlorophyll content is mostly due to impaired functioning of the thylakoid membrane and heavy loss of pigments. Hence, it is a major factor to reduce the yield potential and oil content of mustard. The use of drought-tolerant varieties is a better option to avoid the stress especially in drought-prone areas. Some drought-tolerant varieties are shown in Table 2.

**Table 2.** Various resistant and tolerant varieties of *B. juncea* against different abiotic and biotic stresses.

Stress condition	Recommended varieties	References
Drought	Aravali, Geeta, GM 1, PBR 97, Pusa Bahar, Pusa Bold, RH 781, RH 819, RGN 48, RB 50, Shivani, TM 2, TM 4 and Vaibhav	[26]
Heat stress	Urvashi, RGN 13, RGN 229, Pusa Agrani, Kanti, PM 26, PM 27, DRMR 1165-40, NRCDR 2, NRCD601, PR 20061 (Pant rai 19)	[27]
Frost	RGN-48, RK-9001, RH-8816, RGN-13, RH-819, RH-781, RGN-73 Swarnajyoti	[27] [17]
Salt stress	CS 52, CS 54 and Narendra Rai	[28]
Alternaria blight	PHR 2, RC781, Divya, PAB 9534, EC399301	[7]
White rust	JM-1, JM-2, DMH-1, Basanti, JMWR 08-3	[27] [17]
Powdery mildew	DRMR 150-35, NRCDR 2, NRCDR 601	[27]
Mustard aphids	Glossy B-85, RH 7847, T 6343	[27]

### Nutrient deficiency

Nutrients play a key role in the growth of the crop, as they increase the seed and oil yield by increasing the setting pattern of siliquae on branches, the number of siliquae/plants, and other yield attributes. Among the nutrients, nitrogen is the most responsive nutrient for *B. juncea.*^29^ For its better utilization, it is supplied in two parts –50% as basal and 50% as top dressing after first irrigation, giving higher seed yield under irrigated conditions. It’s requirement to the crop depends on the seed type and organic matter content. Phosphorus is a nutrient that is lacking in many soils but is abundantly absorbed and accumulated by the mustard crops. The total P_2_O_5_ uptake/ton produced ranged between 12.4 and 42.7 kg in mustard.^16^ It has been found that potassium deficiency is a serious issue with mustard and can be overcome by applying 65 kg K_2_O/ha.^30^ It has been shown that sulfur is a vital nutrient for mustard, as its application in combination with balanced amounts of other nutrients significantly increases the oil content (5%–6%) and protein content.^31^ To ameliorate the sulfur deficiency in mustard, 40 kg S/ha is optimal under most field conditions. Generally, mustard is most sensitive to micronutrients deficiency, especially zinc and boron. The concentration of Zn at flowering, pod formation stage, concentration and uptake of Zn in the grain and straw of Indian mustard at maturity increased significantly with increasing Zn levels. Similarly, the seed yield increased significantly (16%–47%) with the application of boron.^16^ In our opinion, despite the use of fertilizers, organic manure can be used to improve overall soil health and reduce evaporation losses of soil moisture. The use of Sestbania green manuring has been found to good for higher mustard yield and improve the soil environment. It has been observed that Foliar spray of BioForce (an organic formulation) 2 mL/L at the time of flowering and siliqua formation stage can enhance the mustard seed yield up to 2059 kg/ha.^16^ Potassium is a key plant nutrient and may be considered to relieve drought, as it regulates turgor pressure, photosynthesis, the translocation of assimilates to various parts of the plant and enzyme activation.

In addition, plant growth regulators (PGRs) may improve stress tolerance in *B. juncea*, as it regulates the germination, formation and distortion of roots and leaves along with stem elongation by the application of gibberellic acid, salicylic acid, and cytokinin. In this context, transcription factors (TFs) are emerging as useful resources for genetic engineering to induce drought tolerance in mustard plants, as they act as major regulators of various stress-regulatory pathways.^27^

### Heat

As global warming is increasing, heat stress has become another major factor that restricts the growth and development of mustard.^7^ *B. juncea* represents higher productivity, when grown under ambient temperature with optimum physio-metabolic processes, but temperature above the ambient temperature may slow down the metabolic process and result in reduced yield. Pollen viability, grain development, fertilization and anthesis time are mostly affected at the flowering and grain-filling stages because these stages are more sensitive to temperature stress.^20^ Due to intensive cropping systems, this stage is delayed and experiences higher temperature (>30 °C) during the seed-filling stage, which hampers the translocation, leading to more shriveled seeds that ultimately reduce the yield of crops. Early sowing of *B. juncea* faces high temperature during the germination stage, leading to a reduction in the plant emergence and poor plant stand. For instance, it has been reported that, in the Central and North-Western plain zones of India, sowing of mustard crops is delayed until the end of November due to late vacation of the Kharif crop (rice), leading to exposure of the crop to high temperature at maturity.^27^

To overcome the crop loss from heat, improved genotypes have been developed in *B. juncea*, which can tolerate high temperature during germination and other growth stages and should be preferred over high-yield varieties. The use of improved varieties by genetic upscaling to thermo-tolerance for late sown mustard is recommended to avoid the heat stress at the maturity/terminal stage for sustainability of oilseed production.

### Frost

It causes significant damage to agriculture and adversely affects the crop yield through altering the morphological, anatomical, and physiochemical characteristics of plants. It is a sudden crop killer, especially in North and North-Eastern regions of China where the temperature suddenly decreases below 0 °C. When the temperature falls below 0 °C, ice crystals form within the intercellular spaces, and the cells undergo dehydration, resulting cell membrane damage and subsequently affecting the physiology of the plant.^32^ Frost affects the yield and grain quality of *B. juncea.* Seeding stage and reproductive stages are highly susceptible to frost in the case of *B. juncea*. Reproductive stages such as gametogenesis, pollination, fertilization, and embryogenesis are affected by low temperature, which leads to poor pollen grains and the formation of few mature seeds. The injury rate of frost stress depends on many important components, such as the duration and amount of cold stress, different stages of plant growth and moisture content. It affects flowering and siliqua development and prevents seed formation, thereby affecting crop productivity, causing considerable yield loss.^33^ Unseasonal frosts during early autumn and late spring significantly reduce the productivity of spring cabbage (*B. oleracea* L.).^34^ It has been reported that xylomannan in the leaf cuticle of *B. napus* provides resistance to ice recrystallization on leaves^35^ however, high stress can cause bleaching or wilting in leaves, which may lead to death.^36^ In addition, seed mortality and seed germination in *B. napus* are affected by low-temperature stress.^37^ It has been observed that low-temperature stress limits the seedling growth during the overwintering of *Brassica napus* L. thereby decreasing the yield in the Yangtze River Basin (YRB) of China. It has been found that deep tillage can promote the growth and safe overwintering of seedlings, It was found that moderate deep tillage (MT) improved rapeseed seedling conditions during overwintering, thus benefiting high-yield acquisition for rapeseed in the YRB region. The use of MT treatment in terms of increased rapeseed production and frost resistance during overwintering has been suggested as a realistic chance of becoming widespread in the future.^32^ In our opinion also establishing a tillage management system for high-yield and highly efficient rapeseed production. Some frost-tolerant varieties are shown in Table 2.

### Salinity

Salt stress is another major limiting factor for the yield potential of mustard. In arid and semi-arid areas, owing to the high surface evaporation rate and poor drainage system, soluble salts get accumulated on the surface of the soil; if the accumulation exceeds the threshold, it adversely affects plant growth and degrades the soil quality. The morpho-physiological and biological processes of mustard, such as growth rates, chlorophyll contents, the leaf area index and flower absorption, are significantly affected due to high salt concentration by unbalancing nutrients and ion contents. Owing to the high concentration of salt content, the osmotic pressure of soil is higher than the root cells, thereby allowing root cells to absorb water from the soil, leading to water and nutrition imbalance in plants, thus adversely affecting plant growth.^38^ Although, moderate salt concentration has been reported to increase the seed biomass in *B. napus,*^39^ high salt concentration negatively affects the seed germination in many *Brassica* species, also showed retardation in plant growth and development, resulting in reduced crop yield and even death of the plant under severe conditions. Various enzymes of *B. juncea*, such as nitrite reductase (NiR), glutamine synthetase (GS), glutamate dehydrogenase (GDH) and asparagine synthetase (ASN), were found to be affected by high levels of salt stress.^40^ Importantly, plants have a natural counter-mechanism to alter stress by modifying their metabolic processes, which may be further enhanced by using thiourea (TU) induction. Recently, some studies have explained the function of TU in inducing salt tolerance in many crop species, including *B. juncea*. This evidence suggests that TU treatment (6.5 mM) improved salt tolerance in *B. juncea* by enhancing the translocation of sucrose from source to sink.^41^ Salt stress can be reduced by cultivating salt-tolerant varieties, as shown in Table 2.

The association of the root endophyte *P. indica* with *B. juncea* can induce salt tolerance in crops by increasing the level of antioxidants in crops.^42^ Foliar application of salicylic acid spray has been shown to improve salt tolerance in *B. napus* through the synthesis of antioxidants and osmolytes for the maintenance of osmotic equilibrium, ROS detoxification, and the facilitation of nutrient and mineral absorption.^43^ Acetic acid treatment has been reported to upregulate salicylic acid along with other signaling molecules, such as hydrogen sulfide and *γ*-aminobutyric acid also the synthesis of osmotic regulation molecules such as trehalose and proline, which help in the regulation of stress against salt as well as lithium.^44^

### Biotic stress

The biotic stress is a critical factor influencing crop growth and development, subsequently affecting the crop yield. Various biotic factors, including fungi, bacteria, pests, and viruses, contribute to yield losses ranging from 50% to 60%.^45^ Fungal infections in the Brassicaceae family are typically caused by four species of Alternaria: *Alternaria alternata, A. brassicae, A. brassicicola*, and *A. raphani*.^46^ Among these, *A. brassicae* primarily infects the oilseed variety Brassicas, while the other three are more commonly associated with vegetable crops.^47^ These pathogens initially cause symptoms in cotyledons at the seedling stage and later affect the foliage, stem, inflorescence and seeds of mature plants. Yield losses attributed to these infections can range from 11% to 100%, depending on the severity of infection, abiotic stress and management factors.^48^ Blackleg disease in the Brassicaceae family is caused by the fungi *Leptosphaeria maculans,* leading to significant economic loss in crop production.^49^ The fungal pathogen *Hyaloperonospora parasitica* causes downy mildew disease in 20 economically important Brassica crops, especially *B. nigra, B. juncea,* and *B. napus*. It is a foliar disease that initially affects cotyledons, followed by true leaves, and manifests as green yellowish spots. Downy mildew is often accompanied by white rust disease and viral diseases, and such combined infections can lead to 23%–55% yield loss.^50^ Sclerotinina stem rot is another fatal disease among *B. napus* and *B. juncea*, caused by the fungus *Sclerotinia sclerotiorum.* The fungi affect the stem and leaves in seedlings, cotyledons and adult plants as well. Hence, they severely affect the yield. The pathogen induces tissue necrosis, water-soaked lesions and sclerotia inside the stem.^51^ Further, *Xanthomonas campestris pv. campestris* (Xcc), the causal agent of black rot, is one of the serious threat to Brassica vegetables worldwide.^52^ This disease is characterized by V-shaped chlorosis and necrosis from the leaf margins, darkening of the leaf mid vein and vascular tissue. Severe infection can lead to systemic wilting and rot. Subsequently, the infection spread through veins to the vascular tissue in the stem and root areas. Soon after the entire plant becomes rot accompanied by secondary soft rot resulting death of the plant.^53^ Soft rot is one of the most common diseases in Brassica, cause by the pathogenic bacteria *Pectobacterium carotovorum*. The bacteria enter the host through wounds or cracks caused by pests or abiotic stress. The development of disease can be visualized as rotting mass tissues.^54^ The clubroot disease is another highly damaging disease among Brassica caused by the protist *Plasmodiophora brassicae.* The general symptoms of the disease include white-colored solid root galls. In severe cases of infection, the symptoms can be visualized as growth stunting, yellowing of leaves, discoloration and rotting of the root gall. Consequently, the water uptake by the plant is impaired, resulting in wilting and, in extreme cases, plant death occur.^55^

Insect pests severely hinder global agricultural productivity. Mustard aphid (*Lipaphis erysimi pseudobrassicae)* is one of the most destructive pests in Brassica, leading to a yield reduction of more than 50%. These aphids feed on plant sap and excrete honeydew, which suppress the plant growth and, on the other hand, creates favorable condition for the proliferation of fungi such as sooty mold.^56^ It has been found that aphids also act as vector for viral diseases. For example, turnip mosaic virus (TuMV) is one of most destructive pathogens transmitted by more than 40 aphid species. This virus causes symptoms such as necrosis, mosaic, vein clearing, plant stunting, and mortality.^57^ In underdeveloped countries, where there is limited access to cost-effective pesticides, the giant white butterfly (*Pieris brassicae*) is a serious threat to crops, potentially resulting in yield loss of up to 90%.^58^ The diamondback moth (*Plutellax ylostella*) is another devastating pest affecting Brassica crops, with the larvae feed on plant foliage causing significant yield loss.^57^ Flea beetle species, primarily, the striped flea beetle (*Phyllotreta striolata*), crucifer flea beetle (*Phyllotreta cruciferae*), and cabbage stem flea beetle (*Psylliodes chrysocephala*) are significant pests affecting Brassica crops globally.^59^ In many parts of Northern India, the striped flea beetle is the dominant flea beetle in Brassica vegetables.^60^ In North America and Europe, the striped flea beetle and crucifer flea beetle are primarily associated with spring oilseed rape, whereas the cabbage stem flea beetle is a major pest in winter oilseed rape.^61^^,^^62^ These beetles colonize and feed on seedlings of plants, resulting in plant crop loss, uneven growth and a reduction in seed yield.^63^ The major pest and diseases that affect Indian mustard are listed in Tables 3 and 4.

**Table 3.** The major pest and diseases of Indian mustard. (Source: Status paper on rapeseed-mustard: National Mission on Oilseed and Oil Palm (NMOOP), Ministry of Agriculture & Farmers Welfare, Govt. of India.).

Pests	Crop stage attacked	Period of activity
**1. Insect**		
i. Mustard aphid (*Lipaphis erysimi*)	Vegetative/flowering and pod formation	December–March
ii. Painted bug (*Bagrada hilaris*)	Leaves	August–October
iii. Tobacco caterpillar (*Spodoptera litura*)	Seedling Maturity stage	October–November March–April
iv. Mustard Sawfly (*Athalia proxima*)	Vegetative	October–December
v. Leafminer (*Chromatomyia horticola*)	Reproductive	February–March
**2. Diseases**		
i. White rust (*Albugo candida*)	November February–March	November February–March
ii. Alternaria leaf spot (*Alternaria brassicae*)	Throughout crop growth	February–March
iii. Powdery Mildew (*Erysiphe cruciferarum*)	Reproductive	February–March
iv. Sclerotina rot (*Sclerotina scleratiarum*)	Vegetative Reproductive	October–November February–March

**Table 4.** Major biotic stresses that cause huge damage to *B. juncea.*

Diseases/Pests	Causal organism	Symptoms	Damage	Management	Yield loss	References
Alternaria blight	*Alternaria brassicae*	Concentric black leaves, stems, and pods.	All developing stages. Qualitative and quantitative determination of seed loss by reducing seed size, seed discoloration and oil content.	Removing weeds and diseased debris Spray of mancozeb (Indofil m-45) @ 2 kg/1000 water at 15 days of interval, 40–45 DAS.	10−70%	[64] [65] [13]
Sclerotinia rot	*Sclerotinia scleroteiorum*	Water-soaked spots on the stem, which were later covered with cottony white growth.	The affected portion of the stem developed a bleached appearance. Girling of the stem results in premature ripening and lodging of the plant.	Use of long-term crop rotation. Control of cruciferous weeds. Application of lime sulfur@ 1 kg/m^2^.	Upto 35%	[66] [67] [13]
White rust	*Albugo candida*	White or yellow pustules with variable sizes & shapes on the lower surface of the leaf.	All parts of the plant except the roots. Both the vegetative and reproductive stages. Severe malformation of inflorescence through hypertrophy and hyperplasia.	Spray of Blitox-50 @ 1.5 kg/ha at 15 d interval. @-3 spray of mancozeb @ 2 kg/ha at 20 d of interval. Seed treatment with Apron 35 SD @ 6 g/kg seeds.	Upto 47%	[64] [65] [13]
Powdery mildew	*Erysiphe crucifararum*	Dirty white, circular, floury patches on either side of the leaves.	Entire leaves, stems, floral parts and pods. Poor seed weight, seed germination and seedling stage.	Seed treatments with iprodione + carbendazim (1:1) @ 2 g/kg seed followed by removal of three lower leaves. ZnSO4 + sulfur as per recommendation was the most effective fungicide for the management of powdery mildew disease.	Upto 18%	[68] [65] [13]
Downy mildew	*Peronospora brassicae*	Yellow & irregular spots on the upper surface & white growth on the lower surface of the leaves. Malformed inflorescence.	Seeds in young plants and leaves and siliqua on adult plants. Reduces photosynthetic activity of plants. Reduce oil content and yield.	Spray 0.2 Zineb at 10-day intervals.	17−37%	[65] [67] [13]
Mustard aphids (pest)	*Lipaphis erysimi*	Curling of infested leaves. Sooty mold grows on honeydew excreted by aphids. Sticky and blighted in appearance	Plant growth, reproduction and development results in yield loss. Insect sucks the sap from leaves, buds and pods. Susceptibility to turnip mosaic disease, as aphid is a vector of this virus.	Crop sown before 20 Oct. can escape the damage. Setup of yellow sticky traps to monitor the population. Conserve the natural enemies such as *Coccinella septempunctata, Menochilus sexmaculata, Hippodamia variegata and Cheilomenes vicina.* *Braconid parasitoid Diaeretiella rapae* is a very active bio-control agent that causes the mummification of aphids. Spray of oxydemeton methyl, dimethoate @625–1000 ml/ha at flowering stage		[66] [13]

### Crop improvement strategies

#### Development of resistant and tolerant varieties

Biotic stress is an important factor that has a significant effect on crop growth and development results in yield loss of mustard. The most economic and ecofriendly way to mitigate the stress is to use resistant and tolerant varieties developed through conventional and molecular breeding approaches. As plants have inherent mechanism for stress survival, various PRRs that sense pathogens or conserved molecules termed pathogen-associated molecular patterns (PAMPs) and then induce PAMP-triggered immunity (PTI), in case of biotic stress. These responses at the molecular, cellular, physiological and biochemical levels enable plants to survive.^69^ Various mechanisms in Brassica species, such as root exudation (e.g., flavonoid glycoside exudation), cell wall composition, callose deposition, and plasma membrane-associated membrane transporters involved in heavy metal flux, can be considered for the development of heavy metal-resistant varieties.^70^ The *B. juncea* cultivars DRMR 150-35, RH 00406, NRCHB 101, Pusa Mustard 27, RLC 3, and RH 725 were reported for their lower aphid resistance index, especially for the mustard aphid *Lipaphis erysimi,*^71^ hence, we propose the use of such varieties for production purpose. Recently, cold tolerance genes such as *BrICE1* and *BrICE2* have been identified in cold-hardy *Brassica* species. These genes are involved in increasing freezing tolerance by enhancing the scavenging of reactive oxygen species (ROS) and regulating the C-repeat/DREB binding factor (CBF) pathway. In our opinion, these cold tolerance genes can be explored more to develop cold-resistant varieties.^72^ We also suggest that pathways associated with the metabolism of galactose, tagatose, glycerone, and fumaric acid can provide valuable insight for the production of drought-tolerant cultivars.^73^ Cytoplasmic male sterility (CMS) lines in Brassica, for instance, *ogu*, *pol*, *nap*, *hau*, *Shaan2A*, and *orf220*, can be potential options for the development of stress-tolerant varieties, as studies have reported that the upregulation of stress-related genes such as monodehydroascorbate reductase, peroxidase, and SOD in CMS lines.^74^^,^^75^

Transgenic plant varieties are being developed for better stress tolerance. Four transgenic lines of *B. juncea* cv Varuna demonstrated significant resistance against *Sclerotinia sclerotiorum.*^76^ Transgenic ***IRT1 B. campestris*** strains have been reported to have improved Cd-stress tolerance.^77^ Molecular techniques such as RNAi-mediated silencing of the vital CHS1 gene of the insect *Plutella xylostella* are also being implemented alongside Bt-transgenic *B. napus* lines to reduce resistance development in the insects.^78^

### Use of biotechnology and nanotechnology

In our opinion, in vitro tissue culture, CRISPR/Cas and RNAi-mediated plant defense and the application of microorganisms will be very useful to develop stress-tolerance cultivars. In this context, tissue culture is considered the most suitable and cost-effective method for the development of stress-tolerant plants. Plants in tissue culture are grown in laboratory conditions under controlled time and space, with a better understanding of the biological and metabolic pathways of plants growing under stressed conditions, which helps to develop a potential stress tolerance in plants. Pusa Jai Kisan is the first high-yielding variety of mustard through tissue culture and is suitable for nontraditional areas.^79^ Recent strategies, such as the use of zinc oxide nanoparticles (ZnO NPs) at optimal concentrations, have been shown to improve callus induction and shoot regeneration in Brassica tissue culture systems.^80^ Furthermore, synergistic application of ZnO NPs with exogenous melatonin has been reported to increase plant tolerance to cobalt-induced stress, thereby enhancing photosynthesis, enzyme activity and nutrient accumulation in *B. napus.*^81^

With rapid advancement in molecular biology and gene editing techniques, CRISPR/Cas has emerged as a prominent tool in crop development. This technique has been successfully applied to enhance resistance against various biotic stresses in crops. CRISPR/Cas-mediated editing has conferred resistance to major pathogens such as *Leptosphaeria maculans* (blackleg disease), *Sclerotinia sclerotiorum* (Sclerotinia stem rot), and *Polerovirus* (Brassica yellows virus) in *B. napus, B. juncea, and B. oleracea*, respectively.^82-84^ In addition to disease resistance, CRISPR/Cas9 has also been employed to improve nutritional quality by reducing glucosinolate biosynthesis and enhancing growth traits, such as increased gibberellin biosynthesis, in *B. oleracea.*^85^^,^^86^ Additionally, higher-yielding varieties of *B. napus* have been developed with increased branching, silique number, and seeds per silique.^87^^,^^88^ RNA interference is a powerful molecular biology tool used to elucidate gene function related to stress resistance and maturity traits, ultimately contributing to yield improvement in Brassica species.^89^ In this regard, the successful use of RNAi to enhance seed oil quality in *B. napus* by targeting the *BnFAE1* gene, resulting in a significant reduction in erucic acid levels and a marked increase in oleic acid content, has been reported.^89^

There are several other actions that can also be utilized to alter biotic stress in crops, such as higher concentration of phenolic compounds, low N contents, increased leaf sugar contents and increased leaf wax deposition, which have been reported to deliver resistance to plants against Alternaria blight disease.^16^ *B. juncea* is susceptible to white rust, whereas *B. carinata* and *B. napus* demonstrated a high degree of resistance. Thus, gene introgression from *B. carinata* and *B. napus* to *B. juncea* through interspecific hybridization can be useful for the development of resistant or tolerant cultivars.^90^

### Improvement of nutrition

*B. juncea* is a nutritionally important source of edible oil. It contains high amounts of erucic acid in its oil content and glucosinolate in meals. The high amount of glucosinolates in meal and erucic acid in oil may create health problems, viz. lipidosis in young animals, fibrosis in older animals, reduced food intake, goiter, stroma and cancer.^91^ In addition, the quality of seeds and their yield must also be improved, which can be accomplished by crop improvement techniques. It is considered one of the most promising technologies for improving seed oil quality, resistance against stresses and for stabilizing the yield of mustard. Various strategies are needed for crop improvement, such as breeding, varietal development, hybrid development, and breeder seed production. In *B. juncea* breeding, considerable emphasis is being placed on the development of low glucosinolate and low erucic acid varieties.^92^ Since 1970, breeding has been ongoing in India to develop varieties with low glucosinolate contents in the seeds up to <30 µ moles/g of defatted cake and <2% erucic acid, termed as double zero (“00”). When a variety contains only one factor, either low erucic (<2%) or glucosinolates (<30 µ mole/g of defatted cake), then the term used is called single zero (“0”). The first low erucic acid (“0”) variety developed in India was LES-39 (Pusa Karishma), followed by LES-1-27 (Pusa Mustard 21), LET-18 (PM 24), and LET-17 (PM-22) in *B. juncea*, whereas the double zero variety was Pusa Double Zero Mustard 31 (PDZM-1).^27^

Varietal development is another strategy that works on genetic enhancement for improving seed and oil yield by developing varieties suitable for early, timely and late sowing conditions to serve the need of different agro-ecological situations of the country, introgression of resistance/tolerance against major biotic (white rust, Alternaria blight, Sclerotinia rot diseases and aphid and painted bug insects) and abiotic stresses (drought, high temperature, frost tolerance and salinity). Under the program, a total of 142 varieties (Indian mustard-91) of rapeseed- mustard were released after the inception of the All India Coordinated Research Project on Rapeseed mustard (AICRP-RM) from 1967 to 2013. By April 2013, ICAR-DRMR have registered fifty novel genetic stocks of rapeseed-mustard including CMS, restorer, low erucic acid & low glucosinolates, high oil content, high oleic acid and low linolenic acid, dwarf, earliness, long main shoot, bold seed, yellow seed, tetra-ocular siliquae, white rust resistance, tolerance to high temperature and salinity during the juvenile stage, high temperature tolerance during the terminal stage and high water use efficiency.^13^

The use of multi-nutrient-based fertilizer (e.g., polyhalite) containing K, S, Mg, Ca, and micronutrients instead of conventional N, P, K, and S-based fertilizers have exhibited better yields and better oil and protein contents in *B. juncea.*^93^ The use of biofertilizers such as the root endophytic fungi *Serendipita indica* co-cultivated with the plant growth-promoting rhizobacteria *Bacillus* or *Enterobacter* has been shown to improve nutrient uptake, auxin production, and fat content in *B. napus.*^94^ Vermi-converted tea industry coal ash can also be a potential alternative to chemical fertilizer for *B. oleracea* var. *Capitata*, as a study, supported improvements in soil quality, nutrient translocation, and crop yield and benefit to cost ratio.^95^

The application of calcium nanoparticles can improve nutrient profile along with drought tolerance, possibly by improving nitrogen metabolism, phytohormone, and flavonoid biosynthesis, as shown in *B. napus.*^96^ Foliar application of zinc oxide and titanium dioxide nanoparticles reported to enhance root and shoot nutrient profile for zinc, iron, manganese, magnesium, calcium, and potassium in *B. napus*.^97^ Seed priming with selenium can also be considered as an eco-friendly approach to manage salt stress, complemented by significant improvement in seed germination, seed biomass, and photosynthetic content.^98^ The flavonoid biosynthetic pathway can also be used for the development of Brassica crop varieties with yellow seed coat, better oil content, and fatty acid composition.^99^

In addition, hybrids provide opportunities to mobilize greater genetic variation and heterosis responses. Generally, hybridization of genetically distinct groups is associated with a higher level of heterosis than hybridization within a group. The development of high-level heterosis in plants requires a large amount of available heterosis, effective pollination control mechanisms and profitability in seed production. It has been reported that the extent of heterosis is 13%–99% in *B. juncea*, 10%–72% in *B. napus*, and 25%–110% in *B. rapa.*^92^ *B. juncea* exploits high levels of heterosis, but owing to its self-pollinating nature, it faces difficulty in seed production. Thus, it is necessary to improve the genetic gain, heterosis and genetic variability of *Brassica juncea* varieties. Mustard hybrids can be developed using five methods: cytoplasmic male sterility (CMS), genetic male sterility, self-incompatibility, chemical hybridizing agents, and genetically engineered male sterility.^100^ The most applicable method for *B. juncea* is the CMS. The large number of CMS systems are well known for Mustard such as *polima, ogura, tournefortii, oxyrrhine, siifolia, trachystoma, moricandia, barthauti, catholica*, *eurocoides,* and *lyratus*. All these sources cannot be used directly because of their negative effects on plant growth and development, such as chlorosis (*ogura, moricandia*), impaired flower opening (*tournefortii, trachystoma*) and the absence of fertility restoration. Successful hybridization using CMS depends upon the availability of efficient fertility restoration. Fertility restorers for *moricandia*, *ogura*, catholica, *erucoides,* and *lyratus* CMS systems could be developed by introgression gene(s) for fertility restoration from cytoplasmic donor wild species.^101^ At present, the Ogura and Moricandia CMS system appears to be the most promising for hybrid development. Sustained efforts resulted in the release of five CMS-based hybrids, out of which NRCHB 506 and DMH 1 were released in 2009, and Coral432 (PAC 432) was released in 2010. DMH 1 is based on a novel CMS system (126 I) developed by the University of Delhi.^102^

### Speed breeding

The prolonged generation time remains one of the primary constraints in conventional breeding programs for the crop production efficiency and often requires decades for the development and improvement of new cultivars.^103^ Breeding approaches such as in vitro culture,^104^ double haploid selection,^105^ marker-assisted selection^106^ and genetic engineering^107^ are being used to reduce the extended breeding cycle. To meet the agricultural demands, speed breeding has emerged as a rapid and more efficient crop improvement strategy wherein crops are cultivated under controlled environmental conditions to induce early flowering and accelerate the breeding cycle.^108^ It has been reported that up to four generations per year can be achieved by speed breeding in *B. napus*, compared to the conventional two to three generations under standard greenhouse conditions.^108^ Up to five generation of *B. napus* under high-temperature, water stress and controlled light conditions.^109^ Hou et al.^110^ reported that the elevated expression of genes in *Brassica rapa* is involved in the biosynthesis of auxin, chlorophyll, ATP, NADPH, and starch, along with the regulation of Rubisco activity under elevated CO_2_ conditions. Controlled environmental condition supplemented with far-red light have been demonstrated to regulate the key genes involved in circadian rhythm, including phytohormones, in *B. alboglabra Bailey*, elevating growth by enhancing the accumulation of photosynthetic pigments and minerals.^111^ Breeder seeds are considered as a backbone of quality seed production and demand-driven programs. It is produced after receiving a declaration from interested public and private stakeholder to further multiply it into foundation and certified seeds and make them available to farmers. Chauhan et al.^112^ analyzed the trend of breeder seed indents of rapeseed-mustard during 25 y, viz., from 1987–1988 to 2011–2012 with the conclusion that indent for Toria was reduced from 35% from 1986–1987 to 1990–1991 to 15% from 2006–2007 to 2011–2012, while intends of Indian mustard increased from 56% to 76% and remain almost the same during the period of 2019–2020. In the last 11 y, the number of breeder seeds produced was 2–3 times higher than indents.^113^ The leading varieties of Indian mustard based on demand for breeder seeds, are Pusa Bold, Varuna, Pusa Jai Kisan, and RH 30.^7^

### Integrated pest management (IPM)

It has been found that mustard is highly vulnerable to a number of pests and diseases. Among these insects, the mustard aphid (*Lipaphis erysimi*) is the key pest of the Brassica crops while sawfly (*Athalia proxima*), painted bug (*Bagrada cruciferarum*) cause yield losses. The yield losses due to aphids may reach 97%, while those due to sawflies may reach 15% and 30%, respectively. The period of peak activity for aphids is January—February in most parts of India.^16^ The integrated pest and disease management strategies include deep ploughing during peak summer months, clean cultivation with regular weeding, and the treatment of seeds with carbendazim 0.1% or thiophanate methyl against seedling diseases and imidacloprid.

Further in case of white rust, the mean disease severity is more than 3%, whereas the application of ridomil MZ 72 WP @ 3 g/l is recommended. When *Alternaria* blight has a disease severity is more than 3%, spraying of Mancozeb 50 WP @ 2 g/l needs to be taken up at 50 and 70 d after sowing. Disease-affected plants should be uprooted and destroyed. Additionally, if powdery mildew is observed, dusting of sulfur@ 1.5 kg/ha or spraying of Sulfex at 2 g/l may be used.

Wherever the ETL level of mustard aphids (per plant) has been crossed, a spray of systemic insecticides, viz. Monocrotophos, Oxydemeton Methyl, etc., may be used at recommended doses. If the population of the beneficial insects in an aphid-infested field is sufficient, insecticide sprays may be avoided.^114^ As soon as the crop begins to turn yellow, it can be harvested and left for drawing. Multiple crop threshers are being used for the threshing of mustard. A moisture content of less than 8% is suggested for storage. Mustard grain can be safely stored in open rooms.^114^

IPM emphasizes the use of cost-effective chemical and biological counterparts to conventional pesticides to reduce the risk to the environment and human health. Resistance to commonly used insecticides such as pyrethroids, carbamates, organochlorines and organophosphates in brassica crop pests in Queensland, Australia, from 1986 to 1992 poses an alarming threat to Brassica crop yield and environmental sustainability.^115^^,^^116^ To address insecticide resistance, IPM strategies implemented in brassica crop fields in Queensland include three main components: (1) improving insecticide application techniques, monitoring pests and including production breaks; (2) adopting genetically modified pest resistance varieties of crops such as *Bacillus thuringiensis var. kurstaki* instead of conventional pesticide use; and (3) advancing research on natural enemies of pests such as parasitoids, enhancing spray coverage and decision-making tools for effective pest management.^117^ In this regard, *Trichoderma* spp. and *Allium sativum* L. has been reported to exhibit biopesticide activity against *S. sclerotiorum* affects *B. juncea.*^118^^,^^119^ Seed treatment with metalaxyl M 31.8 ES at 6 ml/kg and foliar treatment with a combination of mancozeb 68% and metalaxyl 4% at 1.7 g a.i./L seem to be significantly effective in managing white rust in *B. juncea*. Aphid infestation has been shown to be effectively managed with Thiamethoxam 25 WG at 1 g a.i./10 L. Furthermore, integrated treatment involving Thiamethoxam and Trichoderma harzianum applied at 2.5 kg/ha to the soil has demonstrated improved mustard yield.^120^ Neem and endod mix extract reported as potential alternative to synthetic chemical Imidacloprid for aphid management in *B. oleracea var. Capitata.*^121^

### Microbial symbionts

It has been known for a long time that endophytic microbes colonize with plants and live in their roots but that in the last decade, they have value for both increasing crop yield and environmental buffering. Symbiotic interactions between plants and microbes benefit to each other. Endophytic microbes improve plant growth by decreasing pathogenesis and nutrient uptake and by increasing water use efficiency during stress conditions. Some microbes, such as *Pseudomonas* sp. Strain-1 suppresses the sclerotia rot disease, and *Pseudomonas syringae* bacterium induces disease resistance via defense priming in *B. juncea*. Studies have demonstrated that growth-promoting bacteria such as *Enterobacter* and *Bacillus*, along with the root endophytic fungus *Serendipita indica*, enhance physiological parameters, overall growth, and seed yield in *Brassica napus* by facilitating nutrient uptake and stimulating auxin biosynthesis.^94^ Transcriptome analysis of the *B. juncea* microbiome plays a vital role of the endophytic bacteria in regulating plant growth, enhancing photosynthesis, modulating nitrate transporter activity, and maintaining phytohormone signaling and homeostasis.^122^ Furthermore, endophytic bacteria such as *Serratia* and *Arthrobacter* have been shown to increase the organic matter content and enhance the bioavailability of heavy metals in the rhizosphere of *Brassica juncea* growing on vanadium-contaminated soil. In addition, their inoculation improved root morphology, altered heavy metal speciation, and enhanced rhizosphere soil properties and microbial community structure, thereby promoting effective phytoremediation^123^. Hyperaccumulator endophytic bacteria such as *Pantoea agglomerans* and *Bacillus mojavensis* have been shown to promote overall plant growth and development while enhancing the phytoextraction of heavy metals by mitigating oxidative stress. When applied synergistically with biostimulants such as humic acid and biochar, these endophytes further strengthen symbiotic interactions, thereby improving heavy metal uptake efficiency.^124^ The symbiotic association of arbuscular mycorrhizal fungi (AMF) with *Brassica oleracea* in the presence of biochar has been shown to improve soil characteristics such as structure, porosity, and texture. Additionally, this combination enhances phenolic compound accumulation, photosynthetic activity, and overall plant growth.^125^

It has been shown that microbial associations enhance plant tolerance by inducing various stress tolerance pathways in symbiotic plants. For instance, the rhizobacterium *Variovorax* sp. YNA59 has been reported to improve drought tolerance in *B. oleracea* by upregulating the expression of oxidative stress-related enzymes such as superoxide dismutase (SOD), catalase (CAT), and ascorbate peroxidase (APX).^126^ The root endophytic fungus *Serendipita indica*, when associated with Brassica rapa, has been shown to enhance drought tolerance by upregulating drought-responsive genes such as gamma-aminobutyric acid (GABA) in roots and shoots, as well as CAS mRNA in drought-stressed leaves.^127^^,^^128^ *S. indica* also induces salt tolerance in symbiont plant by increasing the production of antioxidant enzymes and plant phytohormones such as gibberellic acid and salicylic acid.^129^ *Enterobacter cloacae* enhances salt tolerance in *B. napus* by promoting the biosynthesis of indole-3-acetic acid (IAA).^130^ In addition to enhancing tolerance to abiotic stress, microbial associations also contribute significantly to resistance against biotic stress. For instance, *Pseudomonas* sp. Strain-1 not only provides protection against Sclerotinia stem rot in mustard but also promotes plant growth by improving nutrient uptake.^131^ Similarly, *Alternaria brassicae* Isolate-D has been shown to confer resistance against Alternaria black spot disease.^132^ Some new approaches, such as integrated nutrient management (INM), are planned to exploit the potential of organic manures, composts, crop residues, agricultural wastes, and biofertilizers and their synergistic effects with chemical fertilizers for increasing the balanced nutrient supply and their use efficiency for increasing the productivity and sustainability of agriculture, and improving soil health and environmental safety. Balanced fertilization with adequate methods also increases the nutrient use efficiency in mustard. The introduction of leguminous crops in the rotational and intercropping sequence via the use of bacterial and algal cultures plays an important role in increasing the nutrient use efficiency. Inoculation of mustard seeds with efficient strains of *Azotobacter* and *Azospirillum* enhanced the seed yield up to 389 and 305 kg, respectively, with 40 kg N/ha. The total NPK uptake was also higher with Azotobacter inoculation. The incorporation of 25% nitrogen through FYM + 75% by chemical fertilizer + 100% sulfur significantly enhanced the uptake efficiency.^16^ Microbial symbiont responses associated with *B. juncea* are summarized in Table 5.

**Table 5.** Important microbial symbionts of *B. juncea* with their responses to crop.

Microbes	Microbial responses to plant	References
*Arbuscular Mycorrhizal Fungi* (AMF)	Enhanced metabolism of plant growth (vegetative stage) Improves absorption capacity of water and nutrients Influence the production of hormones such as auxin and gibberellins. Control biotic stress (powdery mildew up to 80%). Solubilize phosphorus.	[133] [134]
*Achromobacter xylosoxidans strain* Ax10	Significantly improved Cu uptake by plant. Increased the root length, shoot length, and fresh and dry weight of plants.	[135]
*Bacillus edaphicus*	Stimulate plant growth. Facilitates soil Pb mobilization. Enhanced Pb accumulation	[136]
*Pseudomonas sp., Bacillus sp.*	Increase the biomass of the crop. Enhance Ni accumulation in plant tissues.	[135]
*Psychrobacter sp., Bacillus cereus* SRA10	Enhance metal accumulation in plant tissue by facilitating the release of Ni from the insoluble phases in the soil.	[135]
*Bacillus subtilis strain*	Plant growth promoting agent – improve health of plants. Inhibit pathogen of Alternaria blight	[137]
*Trichoderma sp.*	Improve growth during stress Induce protection against oxidative damage. Improve plant health under NaCl-salinity stress.	[138]
*Piriformospora indica*	Improves oil quality by reducing erucic acid and glucosinolates contents. Increase accumulation of N, Ca, Mg, P, K, S, B, Fe and Zn.	[139]

### Integrated omics approach

**Figure 5.**
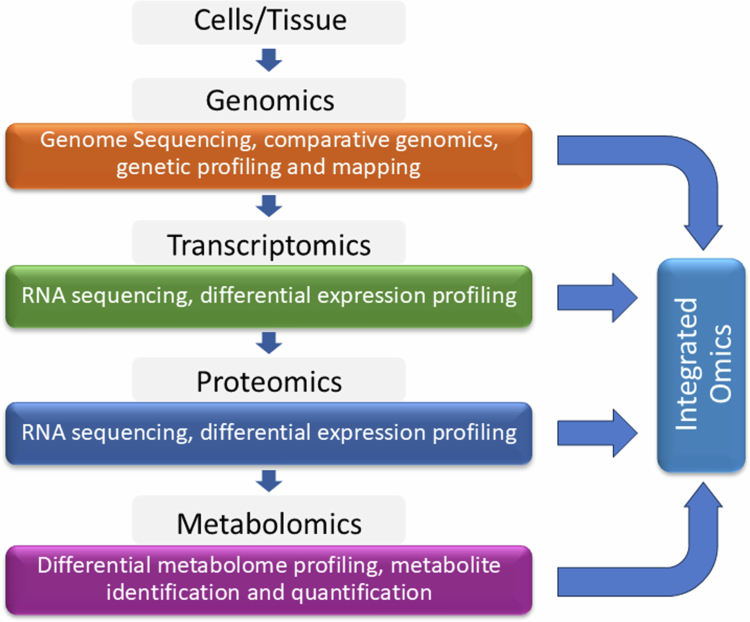
Illustration of omics-based approaches for investigating stress response in crops.

Omics approaches involving genomics, transcriptomics, proteomics, and metabolomics have provided new insights into the genetic improvement of various crops for biotic and abiotic stress resistance (Figure 5). Omics approaches are increasingly applied in Brassica crops to gain insight into the complex mechanisms of host‒pathogen interactions.

In this context, genomics approaches serve as the foundation for crop development through whole-genome sequencing and comparative genomics.^140^ In *B. napus,* the reference genome and pan genomic resources have facilitated the identification of QTLs, SNPs, and genes associated with vernalization, yield, oil quality, efficient nutrient utilization, and tolerance to biotic and abiotic stresses.^141^ Genome-wide association studies (GWAS) are being used to identify genomic markers associated with trait variations, allowing us to understand the precise molecular basis of phenotype variations and thereby helping breeders to develop improved traits with better precision.^142^

Moreover, transcriptomic approaches are better suited than are genomics approaches for complex polyploid crops lacking comprehensive datasets. Associative transcriptomics associate traits with gene expression levels and are being used to identify molecular markers in crops.^143^ Differential expression of genes such as the downregulation of vacuolar storage genes and the upregulation of translocation genes and cell wall integrity maintenance genes elucidates Cd stress-induced adaptations in *B. juncea.*^144^ Integrated transcriptomics and proteomics analysis elucidates the role of genes associated with photosynthesis, antioxidant enzymes, and energy metabolism in cold stress resistance in *B. napus.*^145^

It is important to note that proteomic studies have complemented the genomic and transcriptomic data by providing insights into the translational and post-transcriptional modifications that directly impact the phenotype. These findings revealed stress-induced alterations in protein and cellular functions under stress and during different developmental stages. Mihr et al.^146^ applied a 2-DGE proteomics approach and reported a variation in mitochondrial proteins such as heat shock proteins, NADH dehydrogenase and ATP synthase in the CMS line of *B. napus* line compared with the fertile line.^146^ High-throughput iTRAQ proteomic analysis suggested 833 differentially abundant proteins (DAPs) in the CMS line of *B. oleracea* and 186 DAPs in the CMS line of *B. napus*, respectively, including key proteins involved in protein processing, lipid transfer, sporopollenin synthesis, carbohydrate metabolism, energy metabolism, and programmed cell death.^75^^,^^147^^,^^148^

TMT-based differential proteomic analysis revealed 507 DAPs involved in vernalization in *B. campestris.*^149^ Integrated QTL and proteomics approaches revealed key proteins involved in fatty acid biosynthesis and oil bodies storage that contribute to the higher yield in *B. napus* lines.^150^ Knoch et al.^151^ presented an integrative transcriptomics and proteomics dataset for the development of *B. napus* seeds providing molecular insight to facilitate the production of high oil yielding crops.^151^ Proteomics studies have also been a crucial tool to study the biotic and abiotic stress management in plants (Table 6).

**Table 6.** Proteomics approach to elucidate role of crucial proteins in biotic and abiotic stress responses.

Brassica spp.	Stress factor	Technique used	Crucial differentially abundant proteins (DAPs)	References
*B. rapa*	Freezing stress	LC with tandem MS	Plant hormone signal transduction, alpha-linolenic/linoleic acid metabolism, peroxisome, glutathione metabolism, fatty acid degradation, and secondary metabolite biosynthesis pathways	[152]
*B. campestris*	Cold	RPLC, LC‒MS/MS	Rubisco proteins	[153]
*B. napus*	Arsenic toxicity	iTRAQ proteomics	Antioxidants enzymes	[154]
*B. juncea*	Salt stress	2-DE, MALDI TOF/TOF MS	Osmoregulation, carbohydrate metabolism	[155]
*B. napus*	Salt stress	MALDI TOF/TOF MS	Protein metabolism, damage repair and defense response	[156]
*B. napus*	Salt stress and Exogenous Lipoic Acid	2-DE, MALDI TOF/TOF MS	Photosynthesis, stress defense and signal transduction related proteins	[157]
*B. napus*	Cold	Nano-HPLC-MS/MS	ROS-scavenging proteins	[158]
*B. napus*	Copper	LC‒MS/MS	Chlorophyll biosynthesis, stress-responsive proteins	[159]
*B. juncea*	Cadmium	*2D-DIGE and iTRAQ*	Redox regulation mechanism	[160]
*B. napus*	Drought	LC‒MS/MS	Fatty acid metabolism, increase gibberellic acid and abscisic acid	[161]
*B. oleracea*	salt stress	*2D-DIGE and HPLC-MS/MS*	Proteins involved in xylem differentiation and lignification	[162]
*B. napus*	cadmium stress	LC‒MS/MS	Cell wall modifications, stress/oxidoreductases, and lipid and protein metabolism, defensin-like Cd-binding protein *BnPDFL* in Xylem sap	[163]
*B. rapa*	Cold stress	iTRAQ, LC‒MS/MS	Pentose phosphate pathway, TCA cycle, glyoxylate, dicarboxylate, energy metabolism	[164]
*B. rapa*	Cold stress	iTRAQ, LC‒MS/MS	Biosynthesis of secondary metabolites, oxidative phosphorylation, the pentose phosphate pathway, fructose and mannose metabolism, alpha‒linolenic acid metabolism, ascorbate and aldarate metabolism	[165]
*B. campestris*	High and low temperature	iTRAQ, LC-MS/MS	Redox homeostasis	[166]
*B. rapa*	Cold stress	iTRAQ, LC‒MS/MS	ROS scavenging mechanism and signaling process	[167]
*B. napus*	Cold stress	2-DE, LC‒MS/MS	Chloroplast physiology	[168]
*B. napus*	Cold stress	iTRAQ, LC‒MS/MS	Antioxidant enzymes, photosynthesis, and energy metabolism	[145]
*B. napus*	Heat stress	2-DE	Ascorbate peroxidase	[169]
*B. oleracea*	Heat stress	LC‒MS/MS	Cellular traffic, energy, and metabolism	[170]
*B. campestris*	Heat stress	LC‒MS/MS	Proteins involved in sugar metabolism and the synthesis of osmoprotectants such as glycine betaine and prolin	[166]
*B. napus*	Cadmium stress	LC‒MS/MS	Antioxidant defense system and sulfur assimilation	[171]
*B. **juncea***	Heat stress	MALDI-TOF-MS	DNA repair, signal transduction, and metabolic adaptation	[172]
*B. napus*	Drought stress	2D-DIGE, MALDI-TOF-MS	Downregulation of the chlorophyll content, protein metabolism,	[173]
*B. rapa*	Drought stress	LC MS/MS	Photosynthetic process and glutathione metabolism	[174]
*B. napus*	Salt stress	2DE, MALDI-TOF-MS	Copper/zinc SOD, RuBisCO activase, ferredoxin-NADP reductase, and oxygen-evolving photosystem II	[175]
*B. napus*	Salt stress	LC‒ESI‒MS/MS	Jasmonic acid and abscisic acid	[176]
*B. rapa*	Turnip mosaic virus	iTRAQ-LC-MS/MS	Calcium signaling pathways, non-specific lipid transfer proteins, heat shock proteins, WRKY transcription factors	[177]
*B. oleracea*	*Xanthomonas campestris*	1D nanoflow LC‒MS/MS (Shotgun)	Pathogen-related proteins such as endochitinase	[178]
*B. oleracea*	*X. campestris*	2-DE, MALDI-TOF/TOF MS	Chaperonin, amino acid biosynthesis, ATP synthase, carbohydrate metabolism	[179]
*B. napus*	*Sclerotinia sclerotiorum*	ESI-q-TOF-MS/MS	Protein folding and modifications, hormone signaling, antioxidant defense, proteins involved in photosynthesis and metabolism	[180]
*B. napus*	*X. campestris*	LC‒MS/MS	Redox-related proteins, proteins related to protein degradation	[181]
*B. rapa*	*Plasmodiophora brassicae*	UHPLC-MS/MS	Ubiquitin-26S proteasome, a protein related to the MAPK cascade	[182]
*B. rapa*	*P. brassicae*	iTRAQ proteomics	Proteins involved in the glutathione transferase activity pathway, cytokinin signaling, and arginine biosynthesis pathways	[183]
*B. napus*	*Piriformospora indica*	LC/MS	Metabolic processes, symbiotic signaling, stress/defense responses, energy production, nutrient acquisition	[184]
*B. rapa*	Turnip mosaic virus	iTRAQ, LC-ESI-MS/MS	Ca2+ transporters, heat shock proteins, pathogenesis-related proteins, ROS scavenging proteins	[185]
*B. oleracea*	*Fusarium oxysporum*	LC–MS/MS	Carbohydrate metabolism, Proteins with leucine-rich repeats and legume lectin domains, pathogenesis-related proteins	[186]

In addition, metabolomics has been a powerful tool to bridge the gap between genotype and phenotype and is used to study stress-induced metabolic profiling and metabolic networks in plants.^187^ Metabolic profiling of drought-induced *B. napus* revealed that 17-differential metabolites including tagatose and fumaric acid explaining the role of these metabolites in drought resistance and provide valuable insight into metabolic regulation under drought stress.^73^ Integrative transcriptomics and metabolomics studies highlighted the role of genes and metabolites involved in amino acid and fatty acid metabolism in salt tolerance in *B. napus.*^188^ A different integrative transcriptomics and metabolomics study correlated starch, sucrose and phenylalanine metabolism pathway to the cold stress adaptation in *B. napus.* Therefore, the use of metabolomics and its ability to regulate abiotic stress in rapeseed have been suggested.^189^

## Conclusion

Globally, existing yield of *B. juncea* is much less common than required because of the vast variability in climatic and edaphic conditions. The gap between the demand and actual production of oilseeds can be filled by the selection of appropriate cultivars. The high susceptibility of *B. juncea* for stresses needs to be managed by exploring genetic approaches such as tissue culture, RNAi-mediated plant defense to develop stress-tolerant/resistant cultivars. The continued low productivity of crops offers potential advancements in crop improvement through crop improvement strategies, which, in combination with biotechnological tools can enhance the oil quality and stabilize the yield of *B. juncea*. The microbial symbionts on *B. juncea* are considered as a reliable and eco-friendly approach to improve plant growth under stress conditions by altering their metabolic pathways. *B. juncea* has some potential for the phytoremediation of heavy metals such as lead, mercury, selenium, nickel, etc., which are becoming a serious problem for the environment. In association with microbes, the capacity for phytoremediation is increased. Constant efforts need to be made by conventional crop improvement strategies (discussed above) in combination with biotechnological tools for the development of high-yield varieties with good oil quality and tolerance against biotic and abiotic stresses. In our opinion, integrative omics, including marker-assisted selection (MAS) approaches, integrate multi-omics datasets and provide a holistic understanding from genotype to phenotype through molecular networks. Multi-omics approaches are being used for the better understanding of the adaptation of plants to different stress. Holistic study of resistance against Clubroot in *B.* *oleracea* has been used to develop improved clubroot-resistant varieties.^190^ Plant responses to different nutrient deficiency are better understood at the molecular level with the use of an integrative omics approach.^191^ A comprehensive study of omics at different level helps in the identification of novel genes/proteins and it also highlights the genetic basis of agronomic traits that can be exploited for better/high-yield Brasicca varieties.^192^

In addition, breeding programs must be reoriented to meet the present challenges faced by the crop. Currently, omics breeding has emerged as a novel concept in crop improvement, as it is a more robust and rapid method as compared to conventional breeding. Overall, the integrated omics approach, genome editing, and speed breeding can alter rapeseed production worldwide. The need of the hour is the development of new methodological approaches and crop protection technologies that can be used by farmers in resource-scarce regions to increase the crop yield.
